# Case Report: A primary breast collision tumor composed of myeloid sarcoma and invasive ductal carcinoma

**DOI:** 10.3389/fonc.2026.1788294

**Published:** 2026-03-11

**Authors:** Jia-Sheng Ding, Min Zhang, Fangfang Zhou

**Affiliations:** 1 Department of Intensive Care Unit, Lishui Central Hospital, The Fifth Affiliated Hospital of Wenzhou Medical University, Lishui, Zhejiang, China; 2 Department of Pathology, Lishui Central Hospital, The Fifth Affiliated Hospital of Wenzhou Medical University, Lishui, Zhejiang, China; 3 Department of Ultrasound, Lishui Central Hospital, The Fifth Affiliated Hospital of Wenzhou Medical University, Lishui, Zhejiang, China

**Keywords:** breast, case report, collision carcinoma, invasive ductal carcinoma, myeloid sarcoma

## Abstract

Collision tumors, characterized by the coexistence of distinct malignant neoplasms within the same anatomical site, are rare in the breast. We present a case of a 53-year-old woman with an incidentally discovered palpable mass in the upper inner quadrant of the left breast. Preoperative hematological evaluation was unremarkable. Comprehensive imaging evaluation, including ultrasound, mammography, MRI, and PET-CT, was suggestive of malignancy. A preoperative core needle biopsy was performed but yielded limited material, with pathology suggestive of possible invasive ductal carcinoma, necessitating definitive surgical excision for diagnosis. Following breast-conserving surgery and sentinel lymph node biopsy, histopathological and immunohistochemical analysis revealed a collision tumor composed of myeloid sarcoma (MS) and invasive ductal carcinoma (IDC), the latter exhibiting a triple-negative phenotype (ER-, PR-, HER2-), with no lymph node metastasis. This case highlights the clinicopathological and imaging features of this rare entity and underscores the integral role of multimodal imaging, thorough pathological evaluation, multidisciplinary collaboration, and the limitations of biopsy in heterogeneous lesions in diagnosis and management.

## Introduction

Collision tumors, characterized by the coexistence of two histologically distinct neoplasms within the same anatomical site, are exceedingly rare in breast pathology (1). Instances involving the intersection of hematopoietic malignancies—particularly leukemia—with invasive breast carcinoma are especially scarce in the literature. Documented cases of breast collision tumors to date include invasive ductal carcinoma (IDC) with mucosa-associated lymphoid tissue (MALT) lymphoma (2, 3); breast carcinoma with unspecified lymphoma (4, 5); tubulolobular carcinoma associated with small lymphocytic lymphoma (SLL) (5); cribriform-type IDC with chronic lymphocytic leukemia (CLL) (5); and medullary IDC with CLL (6).

Myeloid sarcoma (MS) is a notably rare extramedullary solid tumor composed of immature myeloid cells. First described by the British physician A. Burns in 1811, its definitive link to acute leukemia was later established by Dock and Warthin in 1902 (7). MS arises from primitive or immature myeloid precursors that infiltrate extramedullary tissues, and it develops in approximately 2% to 14% of patients with acute myeloid leukemia (AML), often accompanying AML, myelodysplastic syndromes, or other myeloproliferative disorders (8).

Herein, we report an exceptional case of a primary breast collision tumor in a patient who presented with a solitary breast mass without evidence of systemic AML. Pathological evaluation revealed the rare coexistence of MS and IDC within the same lesion. This report details the clinical presentation, imaging features, and histopathological characteristics of this unique entity, aiming to expand the understanding of such rare breast tumors and provide a reference for their diagnosis and management.

## Case presentation

A 53-year-old woman presented with an incidentally discovered, painless lump in the upper inner quadrant of her left breast. The lump was described as hard, well-defined, and mobile. She sought medical attention after noting an increase in its size over the preceding week. The patient had no personal history of breast disease and no significant family history of breast pathology.

Physical examination revealed a firm, mobile, non-tender mass measuring 3.0 cm × 3.0 cm in the upper inner quadrant of the left breast. The mass was freely movable without adherence to the overlying skin or deeper tissues. Examination of the right breast was unremarkable. There were no detectable skin changes or palpable lymphadenopathy in either axilla.

**Figure 1:**
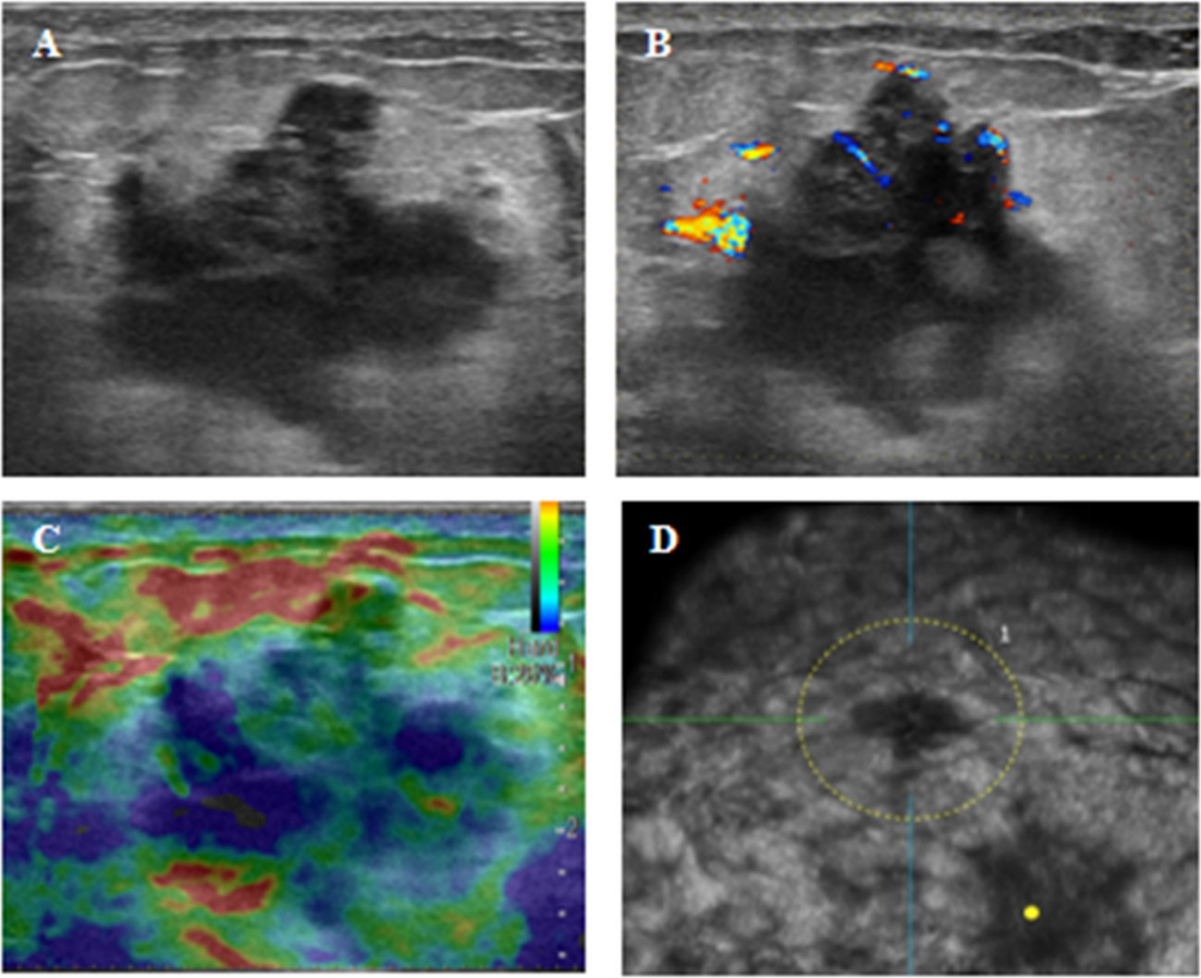
Ultrasound scan and automated breast ultrasound system (ABUS). **(A)** Conventional ultrasound showing a 29×21×20mm hypoechoic mass in the upper inner quadrant of the left breast, with irregular contours and heterogeneous echotexture; **(B)** CDFI displaying Grade II blood flow within the mass; **(C)** Strain elasticity image indicating a moderately firm mass texture; **(D)** ABUS revealing a “Convergence Sign” in the mass.

**Figure 2:**
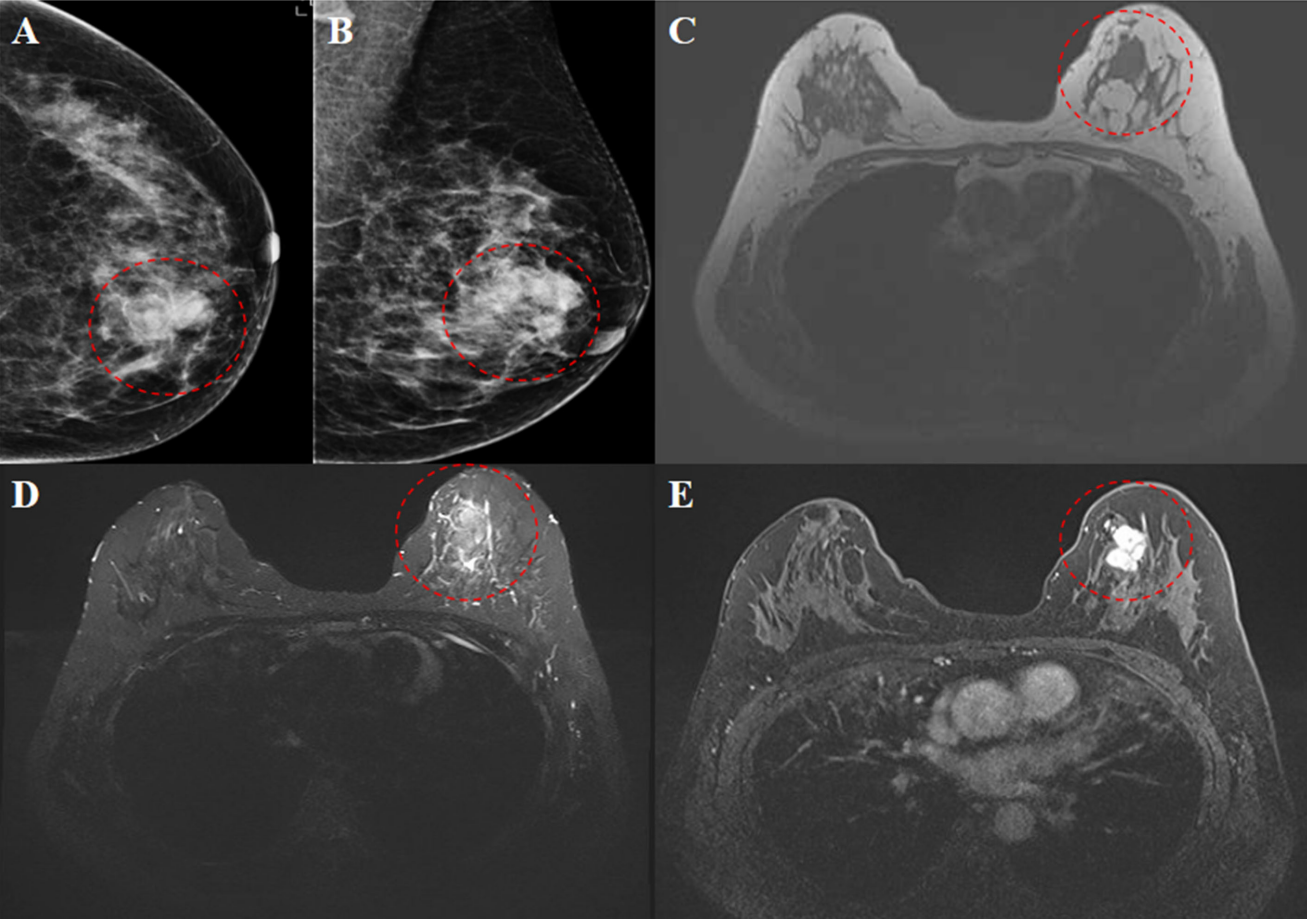
Mammography. High-density mass was identified in the upper inner quadrant of the left breast, characterized by indistinct margins and an irregular shape **(A, B)**. MRI findings revealed low signal intensity on T1-weighted images **(C)** and slightly high signal intensity on T2-weighted images **(D)**. Contrast-enhanced scans demonstrated uneven enhancement and segmented margins **(E)** (indicated by a red dashed circle).

Routine hematological evaluation, including complete blood count (CBC) and peripheral blood smear examination, was performed and revealed no abnormalities, with no evidence of circulating blasts or dysplastic cells. Imaging studies were subsequently performed. Conventional ultrasound identified a 2.9 × 2.1 × 2.0 cm hypoechoic mass in the upper inner quadrant of the left breast. The lesion demonstrated horizontal growth, irregular margins, heterogeneous internal echotexture, and a notable “convergence sign”. Color Doppler Flow Imaging (CDFI) revealed Grade II blood flow, resulting in a BI-RADS category 4C assessment (**Figure 1**). Mammography showed a slightly high-density, irregular mass measuring 2.4 × 1.9 cm, which was also classified as BI-RADS 4C (**Figures 2A, B**). Breast magnetic resonance imaging (MRI) demonstrated a lesion with low signal intensity on T1-weighted images and slightly high signal intensity on T2-weighted images. Post-contrast images revealed heterogeneous enhancement with segmented margins, features indicative of malignancy and warranting a BI-RADS 5 classification (**Figures 2C–E**). Positron emission tomography-computed tomography (PET-CT) confirmed no abnormal fluorodeoxyglucose (FDG) uptake beyond the known breast lesion, indicating no evidence of distant metastasis.

Given the highly suspicious imaging features, an ultrasound-guided core needle biopsy was performed. However, the obtained specimen was deemed limited and suboptimal for definitive diagnosis. Pathological assessment of the biopsy sample raised the possibility of invasive ductal carcinoma but was inconclusive due to scant cellularity and sampling issues. This finding, combined with the high clinical and radiological suspicion of malignancy, was reviewed at our institution’s multidisciplinary tumor board (MDT). The board recommended proceeding directly to therapeutic surgical excision (lumpectomy with sentinel lymph node biopsy) to obtain a complete specimen for accurate diagnosis and treatment. The patient underwent breast-conserving surgery along with sentinel lymph node biopsy. Intraoperatively, a solid mass measuring 3 cm × 2 cm with irregular shape and indistinct borders was excised from the left upper inner quadrant. Intraoperative frozen section analysis was suggestive of a myeloid sarcoma, prompting a comprehensive immunohistochemical workup for definitive characterization.

**Figure 3:**
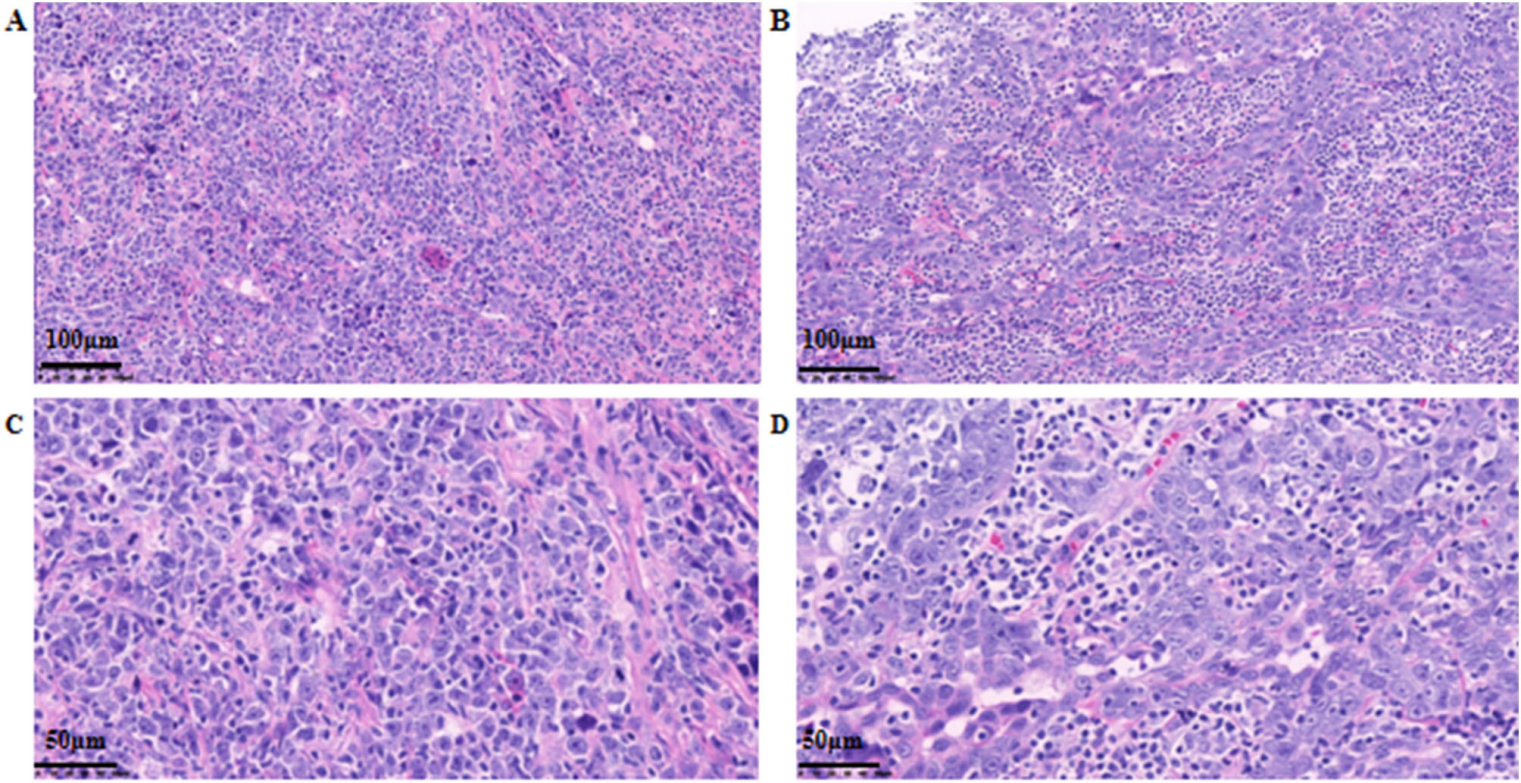
Histopathological findings. **(A)** Hematoxylin-eosin (HE) staining in the area of myeloid sarcoma (×200); **(B)** HE staining in the area of invasive ductal carcinoma (×200); **(C)** HE staining in the area of myeloid sarcoma (×400); **(D)** HE staining in the area of invasive ductal carcinoma (×400).

**Figure 4:**
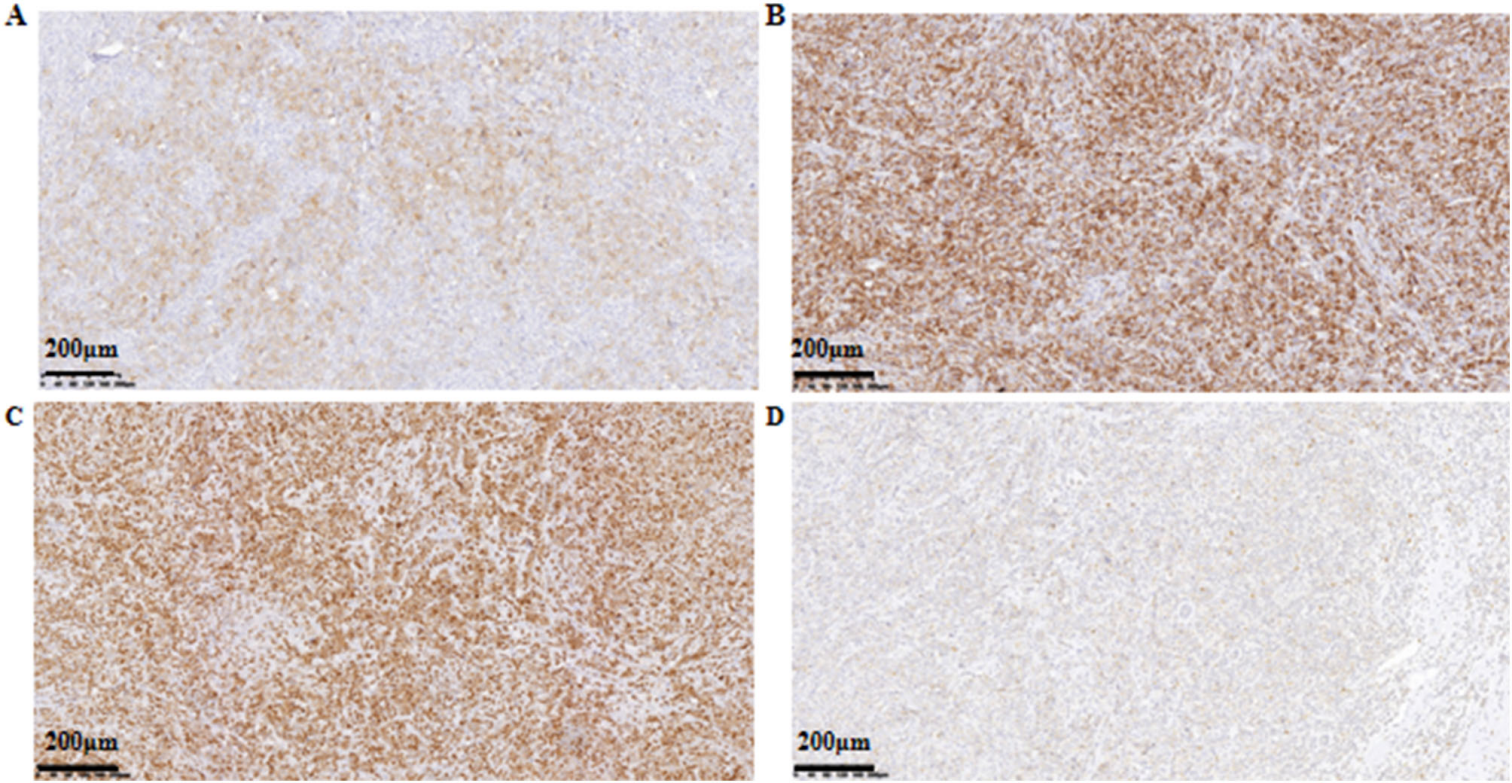
Immunohistochemical staining (× 100). **(A)** Immunostaining for CD117; **(B)** Immunostaining for CD43; **(C)** Immunostaining for CD15; **(D)** Immunostaining for MPO.

Histopathological examination of the resection specimen revealed a tumor measuring 3 cm × 1.6 cm × 1.6 cm. Microscopically, the tumor was biphasic: one component consisted of a diffuse proliferation of medium to large atypical cells within a fibrous stroma. These cells exhibited pale cytoplasm, delicate chromatin, and conspicuous nucleoli, consistent with a myeloid sarcoma. The second component comprised focal areas of high-grade (grade III) invasive ductal carcinoma, with the largest focus measuring approximately 0.2 cm in diameter (**Figure 3**). Immunohistochemical analysis of the sarcomatous component showed positivity for Leukocyte Common Antigen (LCA), Myeloperoxidase (MPO), CD117, CD15, and CD43. The Ki-67 proliferation index was markedly elevated at approximately 75% (**Figure 4**). The invasive ductal carcinoma component was negative for estrogen receptor (ER), progesterone receptor (PR), and HER2 (score 0), confirming a triple-negative phenotype. The Ki-67 proliferation index within the carcinoma component was approximately 40%. No lymphovascular invasion was identified. All surgical margins (superior, inferior, medial, lateral, and basal) were negative for both carcinoma and sarcoma. The final integrated diagnosis was a collision tumor composed of myeloid sarcoma and invasive ductal carcinoma. The pathological stage was pT1aN0M0 (based on the 0.2 cm invasive ductal carcinoma component), with no lymph node involvement. A postoperative bone marrow biopsy from the iliac crest showed no morphologic evidence of hematologic malignancy or significant dysplasia, confirming the diagnosis of primary (isolated) myeloid sarcoma. Despite recommendations for adjuvant chemotherapy following an acute myeloid leukemia (AML)-type regimen, the patient declined this treatment. She also declined scheduled follow-up examinations. A telephone follow-up conducted three years after surgery confirmed that the patient was alive; however, detailed information regarding her current clinical status or any evidence of disease recurrence was not available.

## Discussion

Collision tumors are rare pathological entities, predominantly documented in isolated case reports. Histologically complex tumors of this type are categorized into two clinicopathological groups: collision tumors and composite tumors. Composite tumors lack distinct histological boundaries and often display intermixed features (9). In contrast, collision tumors consist of two or more histologically distinct malignant neoplasms—such as carcinoma-carcinoma or carcinoma-sarcoma combinations—that coexist within the same anatomical site, separated by non-neoplastic stroma, each maintaining its own morphological and immunophenotypic identity (10). Such tumors frequently occur at epithelial transition zones, including the gastroesophageal junction, cervix, and anorectal region, with the combination of squamous cell carcinoma and adenocarcinoma being the most commonly reported. Other documented combinations include adenocarcinoma with sarcoma or lymphoma (11).

This case presents an exceptionally rare breast collision tumor composed of myeloid sarcoma (MS) and invasive ductal carcinoma (IDC). Myeloid sarcoma is a solid extramedullary tumor of immature myeloid cells that may occur in association with acute myeloid leukemia (AML) or myelodysplastic syndromes, or rarely as an isolated primary lesion without bone marrow involvement. It is recognized as a distinct entity in the World Health Organization classification of hematopoietic and lymphoid tumors (12). MS is reported in approximately 2.5%–9.1% of AML cases and may appear before, during, or after the diagnosis of systemic leukemia. Primary or isolated MS—defined by the absence of concurrent bone marrow or systemic disease—is particularly uncommon (8). In our case, the diagnosis of primary breast MS was established following normal bone marrow aspiration and the absence of extramedullary uptake on PET-CT. The patient’s age (53 years) falls within the reported range for primary MS (16–73 years) (12), and her presentation with a painless breast mass is consistent with prior descriptions (13).

Histologically, MS typically exhibits a diffuse proliferation of medium-to-large immature cells with fine chromatin, moderately pale cytoplasm, and variably prominent nucleoli. Myeloperoxidase (MPO) is considered the most sensitive and specific immunohistochemical marker, showing positivity in 77%–97% of cases (14). Other supportive markers include lysozyme, CD68, CD117, CD43, and CD15. The Ki-67 proliferation index is characteristically high, ranging from 50% to 95% (15). In our case, tumor cells expressed LCA, MPO, CD117, CD43, and CD15, with a Ki-67 index of approximately 75%, supporting the diagnosis. This underscores the necessity of employing a comprehensive panel of immunohistochemical stains for accurate diagnosis. The IDC component was identified as triple-negative, a subtype known for its aggressive biological behavior, which adds further complexity to the prognostic assessment of this collision tumor.

Imaging features of breast MS are not well-defined due to its rarity. Reported MRI characteristics may include slightly high signal on T1-weighted images (reflecting high protein content), higher peripheral signal on T2-weighted or FLAIR sequences, restricted diffusion on DWI, and homogeneous enhancement (16). Tumors often appear relatively homogeneous and may demonstrate cystic necrosis or perilesional edema when large (17). In our patient, MRI findings—low T1 signal, slightly high T2 signal, restricted diffusion, and heterogeneous enhancement—deviated from typical descriptions, likely attributable to the collision nature of the lesion with admixed epithelial and myeloid components.

On ultrasound, breast MS generally presents as a hypoechoic mass with variable margins, lacking specific features (18). PET-CT, although not routine in leukemia workup, is valuable in MS for staging and distinguishing primary from secondary involvement (19). In this case, ultrasound showed an irregular, hypoechoic mass with heterogeneous echotexture, while PET-CT revealed focally increased FDG uptake (SUVmax 7.1) without evidence of distant disease. Given its nonspecific imaging appearance, MS should be considered in the differential diagnosis of breast masses even in patients without known hematologic disorders.

This case illustrates a significant diagnostic pitfall: the limitation of core needle biopsy in accurately characterizing heterogeneous or collision tumors. The preoperative biopsy, yielding limited material, suggested possible IDC but completely missed the MS component. This discrepancy highlights that biopsy sampling may not be representative of the entire tumor mass, especially in neoplasms with distinct, spatially separate components. The inherent sampling error and tumor heterogeneity can lead to incomplete or misleading diagnoses, emphasizing that surgical excision with comprehensive pathological examination remains the gold standard for definitive diagnosis in complex breast lesions.

Primary breast MS is generally associated with a more favorable prognosis than systemic AML. However, untreated primary MS may progress to AML within months to years, emphasizing the importance of timely diagnosis and intervention (20). Although no standardized therapeutic protocol exists, treatment typically follows AML-type chemotherapy regimens, often combined with local surgery. In the present case, the patient declined adjuvant chemotherapy. This decision presents a therapeutic challenge, particularly regarding local control. While adjuvant chemotherapy is paramount for systemic control of the MS component, the role of adjuvant radiotherapy (RT) for local-regional control in such collision tumors warrants consideration. For the triple-negative IDC component (grade III, pT1a, margin-negative, LVI-negative), adjuvant RT after breast-conserving surgery is standard to reduce local recurrence. For the MS component, RT can be effective in controlling isolated extramedullary disease. Although not utilized in our case, adjuvant RT could represent a crucial consolidative treatment option, especially when systemic therapy is refused or contraindicated. This approach finds parallels in the management of other rare, non-chemotherapy-sensitive breast tumors where local control is paramount. For instance, in malignant phyllodes tumors, adjuvant RT has been shown to significantly improve local control, particularly in cases with close surgical margins (21). A telephone follow-up at three years confirmed survival, though detailed clinical and recurrence data were unavailable. The lack of structured follow-up and the patient’s refusal of adjuvant therapy are significant limitations of this report, precluding definitive conclusions about long-term outcomes and the efficacy of the surgical intervention alone.

## Conclusion

We report a rare case of a primary breast collision tumor composed of myeloid sarcoma and invasive ductal carcinoma. It underscores the diagnostic challenge posed by such tumors, including the potential for preoperative biopsy to yield incomplete or misleading results due to sampling limitations. This case emphasizes the critical role of comprehensive immunohistochemical analysis and multidisciplinary discussion in accurately diagnosing such complex breast neoplasms. It enriches the spectrum of primary breast tumors and highlights the necessity for clinicians and pathologists to consider collision tumors in differential diagnosis to inform appropriate clinical management. The therapeutic dilemma posed by the patient’s refusal of chemotherapy underscores the need for further discussion and research into the potential role of adjuvant radiotherapy in ensuring local control for these rare entities.

## Data Availability

The original contributions presented in the study are included in the article/supplementary material. Further inquiries can be directed to the corresponding author.

## References

[1] Jafarian N Kuppler K Rosa M Hoover S Patel B . Chronic lymphocytic leukemia and invasive ductal carcinoma presenting as a collision breast tumor. Clin Breast Cancer. (2015) 15:e209–12. doi: 10.1016/j.clbc.2015.02.001, PMID: 25818398

[2] Quilon JM Gaskin TA Ludwig AS Alley C . Collision tumor: invasive ductal carcinoma in association with mucosa-associated lymphoid tissue (MALT) lymphoma in the same breast. South Med J. (2006) 99:164–7. doi: 10.1097/01.smj.0000198640.58397.c5, PMID: 16509555

[3] Susnik B Rowe J Redlich PN Epstein HD Bhargava R Wang Z . A unique collision tumor in breast: invasive ductal carcinoma and mucosa-associated lymphoid tissue lymphoma. Arch Pathol Lab Med. (2004) 128:99–101. doi: 10.5858/2004-128-99-AUCTIB, PMID: 14692838

[4] Wiernik PH Hu X Ratech H Fineberg S Marino P Schleider MA . Non-Hodgkin’s lymphoma in women with breast cancer. Cancer J. (2000) 6:336–42. 11079174

[5] Cheung KJ Tam W Chuang E Osborne MP . Concurrent invasive ductal carcinoma and chronic lymphocytic leukemia manifesting as a collision tumor in breast. Breast J. (2007) 13:413–7. doi: 10.1111/j.1524-4741.2007.00451.x, PMID: 17593048

[6] Catteau X Dehou MF Dargent JL Hackx M Noel JC . Chronic lymphocytic leukemia mimicking recurrent carcinoma of the breast: case report and review of the literature. Pathol Res Pract. (2011) 207:514–7. doi: 10.1016/j.prp.2011.05.007, PMID: 21689894

[7] Zhao H Dong Z Wan D Cao W Xing H Liu Z . Clinical characteristics, treatment, and prognosis of 118 cases of myeloid sarcoma. Sci Rep. (2022) 12:6752. doi: 10.1038/s41598-022-10831-7, PMID: 35474239 PMC9042854

[8] Almond LM Charalampakis M Ford SJ Gourevitch D Desai A . Myeloid sarcoma: presentation, diagnosis, and treatment. Clin Lymphoma Myeloma Leuk. (2017) 17:263–7. doi: 10.1016/j.clml.2017.02.027, PMID: 28342811

[9] Shin YD Lee SK Kim KS Park MJ Kim JH Yim HS . Collision tumor with inflammatory breast carcinoma and Malignant phyllodes tumor: a case report and literature review. World J Surg Oncol. (2014) 12:5. doi: 10.1186/1477-7819-12-5, PMID: 24400686 PMC3895737

[10] Cohen PR Calame A . Collision tumors are multiple skin neoplasms at one site (MUSK IN A NEST): a new paradigm for their terminology and classification. Int J Dermatol. (2023) 62:e242–e3. doi: 10.1111/ijd.16144, PMID: 35239975

[11] Ben Khoud M Ingegnere T Quesnel B Mitra S Brinster C . Acute myeloid leukemia: is it T time? Cancers (Basel). (2021) 13:2385. doi: 10.3390/cancers13102385, PMID: 34069204 PMC8156992

[12] Choi SM O’Malley DP . Diagnostically relevant updates to the 2017 WHO classification of lymphoid neoplasms. Ann Diagn Pathol. (2018) 37:67–74. doi: 10.1016/j.anndiagpath.2018.09.011, PMID: 30308438

[13] Pace M Guadagno E Russo D Gencarelli A Carlea A Di Spiezio A . Myeloid sarcoma of the breast as blast phase of JAK2-mutated (Val617Phe exon 14p) essential thrombocythemia: A case report and a systematic literature review. Pathobiology. (2023) 90:123–30. doi: 10.1159/000525163, PMID: 35850113

[14] Tran VT Phan TT Mac HP Tran TT Ho TT Pho SP . The diagnostic power of CD117, CD13, CD56, CD64, and MPO in rapid screening acute promyelocytic leukemia. BMC Res Notes. (2020) 13:394. doi: 10.1186/s13104-020-05235-7, PMID: 32847610 PMC7449061

[15] Kavus H Ding Y Dhesi M . Updates in immunohistochemistry for hematopoietic and lymphoid neoplasms. Arch Pathol Lab Med. (2024) 148:e25–40. doi: 10.5858/arpa.2022-0465-RA, PMID: 37270801

[16] Seok JH Park J Kim SK Choi JE Kim CC . Granulocytic sarcoma of the spine: MRI and clinical review. AJR Am J Roentgenol. (2010) 194:485–9. doi: 10.2214/AJR.09.3086, PMID: 20093613

[17] Meyer HJ Pönisch W Schmidt SA Wienbeck S Braulke F Schramm D . Clinical and imaging features of myeloid sarcoma: a German multicenter study. BMC Cancer. (2019) 19:1150. doi: 10.1186/s12885-019-6357-y, PMID: 31775680 PMC6882227

[18] Kawamoto K Miyoshi H Yoshida N Takizawa J Sone H Ohshima K . Clinicopathological, cytogenetic, and prognostic analysis of 131 myeloid sarcoma patients. Am J Surg Pathol. (2016) 40:1473–83. doi: 10.1097/PAS.0000000000000727, PMID: 27631510

[19] Aschoff P Hantschel M Oksuz M Werner MK Lichy M Vogel W . Integrated FDG-PET/CT for detection, therapy monitoring and follow-up of granulocytic sarcoma. Initial results. Nuklearmedizin. (2009) 48:185–91. doi: 10.3413/nukmed-0236, PMID: 19710955

[20] Goyal G Bartley AC Patnaik MM Litzow MR Al-Kali A Go RS . Clinical features and outcomes of extramedullary myeloid sarcoma in the United States: analysis using a national data set. Blood Cancer J. (2017) 7:e592. doi: 10.1038/bcj.2017.79, PMID: 28841208 PMC5596389

[21] Liscu HD Ionescu AI Mologani I Verga N . Phyllodes tumor of the breast: A case report regarding the importance of fast interdisciplinary management. Rep (MDPI). (2025) 8. doi: 10.3390/reports8010017, PMID: 40729230 PMC12199950

